# Investigating factors affecting the evaluation of teachers’ medical universities from the students’ point of view: a systematic review

**DOI:** 10.1186/s12909-024-05161-3

**Published:** 2024-02-23

**Authors:** Zahra Sooki, Khadijeh Sharifi, Forough Faroughi

**Affiliations:** 1 Trauma Nursing Research Center, Faculty of Nursing and Midwifery, Kashan University of Medical Sciences, Kashan, Iran; 2 Student Research Committee, Kashan University of Medical Sciences, Kashan, Iran

**Keywords:** Evaluation, Assessment, Teachers, Students, University, Systematic review

## Abstract

**Background:**

Faculty evaluation is essential as a principle in educational organizations because it helps measure the quantity and quality of education in universities and educational institutions. There are various ambiguities regarding the desirable and deserving characteristics of a good teacher. Therefore, this study was conducted with the aim of investigating factors affecting the evaluation of teachers’ medical universities from the perspective of students.

**Methods:**

A systematic review study was conducted by searching for studies in both Persian and English languages from 2014 to 2022 in the following databases: Pubmed, Web of Science, Scopus, Cochrane, Google Scholar, and ScienceDirect, Magiran, SID, Iran Doc using keywords including Evaluation, Assessment, Estimate, Appraisement, Appraisal, Faculty Member, Professor, University, and College, as well as their MeSH equivalents, using “AND” and “OR” operators. The results of the articles about investigating factors affecting the evaluation of teachers’ medical universities from the perspective of students were reviewed, summarized, and reported.

**Results:**

In the initial search, 3949 articles were found, and after evaluation, finally 21 articles were included in the systematic review. Based on the findings, investigating factors affecting the evaluation of teachers’ medical universities from the perspective of 130,187 students can be categorized into 6 dimensions and 53 components. These dimensions include individual and professional characteristics of the educational system, attitude within the educational system, educational programs and guides, teaching methodology, internal coherence of educational resources, and evaluation system information.

**Conclusion:**

The results of the articles about investigating factors affecting the evaluation of teachers’ medical universities from the perspective of students were reviewed, summarized, and reported. It is necessary to pay attention to the factors affecting the evaluation of teachers in the recruitment of faculty members. Additionally, by holding practical training workshops with consideration of various dimensions that have an impact on faculty evaluation and student learning, it is possible to enhance the expertise of faculty members.

**Supplementary Information:**

The online version contains supplementary material available at 10.1186/s12909-024-05161-3.

## Introduction

The core of education is teaching and learning, and learning takes place best when there are effective teachers. One of the ways to determine the effectiveness of teaching is through students’ evaluation of teachers and surveys that students complete each academic term [1]. Evaluation is a pervasive and essential process that is recognized as a principle in educational organizations [2, 3]. Because evaluation helps to measure the quantity and quality of education in universities and educational institutions. University teachers are considered the main pillars of education in the modern educational system’s structure and framework, and their performance plays a critical role in the overall effectiveness of the education system [4]. Faculty evaluation is one of the most complicated types of evaluation, and its complexity is due to the lack of credibility and accuracy of the tools and measurement methods used. Therefore, it is suggested that different aspects of evaluation should be considered for the final judgment, taking into account various evaluation criteria [5].

However, there are various ambiguities about the desirable and deserving characteristics of a good teacher. Some believe that a desirable teacher is someone who has expertise in their field of study and can provide high levels of knowledge and expertise in related areas. Others believe that the knowledge and skills should be practical and applicable. Some also consider the ability to cultivate ethical matters in students and the role of guidance to be important, and in fact, they have given the highest score to the personal and ethical characteristics of the teacher [6]. Nobakht et al., 2013 also was shown that students rated the control and management methods of the class, the personal and social appearance of teachers, and the mutual relationships between teachers and students as the most important factors [7]. In another study, 94% of students considered teachers scientific mastery of the subject matter, 91% of students, teachers expression power, and 90%, teachers efforts in clarifying scientific concepts as essential evaluation criteria for teachers [8]. Studies have shown the existence of gender bias in students’ evaluations of teaching [9, 10]. Some studies have reported that students’ criteria for judging their teachers can be different from their actual teaching quality [11]. The results of some research showed a positive relationship, when teacher was knowledgeable, friendly, and fair from the students’ point of view, higher evaluation scores were reported [12]. The main problem of the evaluation systems used in universities is that it only takes into account some specific aspects of teaching, including the transparency and ethics of teachers, which cannot accurately reflect the quality of teaching and learning [13]. Understanding students’ opinions about factors affecting the evaluation of teachers can provide useful guidance for addressing existing problems. Additionally, identifying strengths and weaknesses in the evaluation of teachers can help educational planners develop a program to improve and enhance the quality of evaluation. Considering the various factors discussed in the studies, also the existence of some contradictions in these studies and the ambiguities in this regard, this study was conducted to investigate factors affecting the evaluation of teachers’ medical universities. Researchers in order to answer the research question, what are the factors affecting the evaluation of teachers’ medical universities from the students’ point of view? They conducted a systematic review with the aim of investigating factors affecting the evaluation of teachers’ medical universities from the students’ point of view.

## Methods

### Systematic review protocol

A systematic review was conducted based on a predesigned protocol in accordance with the Preferred Reporting Items for Systematic Reviews and Meta-Analyses (PRISMA) statement [14].

### Search strategy

During the period of January 2014 to June 2022, a search was conducted in databases and search engines, including Pubmed, Web of Science, Scopus, Cochrane, Google Scholar, and ScienceDirect, Magiran, SID, Iran Doc using keywords such as evaluation, assessment, estimate appraisal, appraisal faculty member, professor, university, college, as well as their MeSH equivalents, using the “AND” and “OR” operators.

### Inclusion and exclusion criteria

The present study is designed to answer the question of investigating factors affecting the evaluation of teachers’ medical universities from the perspective of students. The inclusion criteria for articles include being available, published in reputable research and university journals, including descriptive, observational, and qualitative studies, the presence of keywords or their equivalents in the title or abstract, and articles in both English and Persian. The exclusion criteria include articles that did not address evaluation in universities and did not focus on teachers in medical sciences.

### Study selection, data extraction and study quality

**Fig. 1:**
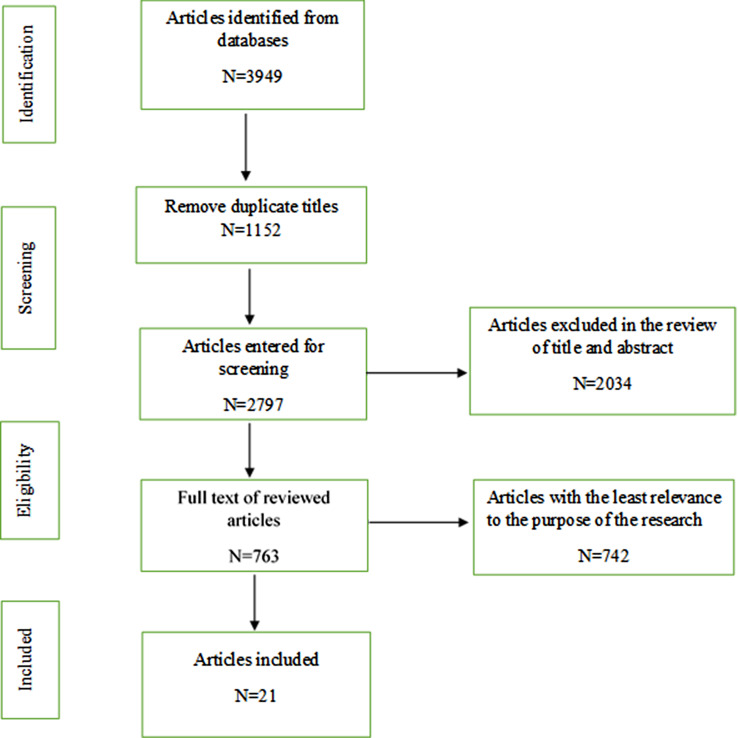
PRISMA flow diagram of the study selection process

Figure 1 depicts the study selection and review processes. During the search process, a total of 3,949 articles were found. After removing duplicates and reviewing the titles, abstracts, and full texts, 21 studies were ultimately included in the analysis. The references of the final articles were also reviewed. The STROBE and COREQ checklists were used to evaluate the quality of articles. The STROBE checklist contains 22 items and 34 sub-items, 20 articles were evaluated by this checklist. Scores of 0–1 and 2 were given for each sub-item based on its correctness, uncertainty, or incorrectness. The maximum and minimum scores obtained were 48 and 34, respectively. To evaluate the quality of a qualitative article, the COREQ checklist, which contains 32 items, was used. Scores of 0 and 1 were given based on reporting or non-reporting of each item, and the article’s score was 13. Data extraction was performed using a checklist including author information, publication year, study objective, study design, sample size, data collection method, and results. The results obtained from the analysis of the articles were summarized and reported. All search, review, and quality assessment steps were performed by two researchers (F.F and Z.S) and in cases of disagreement, a third researcher (Kh.Sh) was consulted. To access the proposal of this study, you can contact the corresponding author.

## Results

The search yielded 3949 articles from databases. After removing duplicates, 2797 articles remained; their titles and abstracts were scanned, and 763 relevant articles were identified. The full texts of these 763 articles were reviewed. Among the included articles, 21 original articles were undergone further data extraction and analysis. The key information of the 21 original articles is summarized in Table 1.

**Table 1:** Studies included in the analysis process regarding factors affecting the evaluation of teachers’ medical universities from the students’ point of view

Row	Author’s name year	Country	Type of study	The purpose of the study	Number of samples /sampling/ tools	The most important findings
1	Vahabi. A et al. 2015 [15]	Iran	cross-sectional study	Determine the factors affecting teacher evaluation scores from the viewpoint of the students in Kurdistan University of Medical Sciences.	384 students were randomly selected, the data collection tool was a questionnaire including demographic questions and factors affecting the teachers evaluation from the students’ point of view.	• The most important factors affecting the scores of teachers’ evaluation from the viewpoint of the students were: teachers’ knowledge on the subject matter (4.53 ± 0.8 out of 5), teachers’ ability to convey the lessons (4.52 ± 0.78), compatibility of class content material and final exam questions (4.40 ± 0.79) • The least important of these factors were gender (3.48 ± 1.01), teachers’ age (3.28 ± 1.14) and conducting tests to measure academic achievement of faculty members (3.1 ± 1.42).
2	López-Cámara. AB et al. 2015 [16]	Spain	descriptive and correlational	Discovering competency-based dimensions that evaluate the teaching quality of university professors from the students’ point of view and determining competency-based factors that determine the quality of teaching in each of the extracted dimensions.	1316 students, the teacher’s teaching quality evaluation questionnaire, approved by the validation experts of 32 Spanish public universities, using the Delphi technique	• The students believed that the keys to evaluating a professor’s teaching activity include these items; teaching method, course guide design (theoretical and practical), professors’ attitude, internal coherence of educational resources, information about evaluation systems
3	Yaminfirooz. M et al. 2017 [4]	Iran	cross-sectional descriptive-analytic study	identify the most important criteria which were used to assess professors by their students.	Studying all fields and degrees of Babol University of Medical Sciences (315 students). Data Collection tools; Vahabi et al.‘s questionnaire includes 24 questions in four areas: personal characteristics, teaching skills, educational rules, and communication skills.	• Among the 24 examined criteria, the teacher’s mastery of the subject, the ability to understand the course material (both with an average of 4.83 ± 0.40), the ability to communicate with students (4.54 ± 0.62), compliance with the content The academic level of the learners with an average of (4.50 ± 0.61) was the most important criterion for the students in evaluating the professors, • Some criteria such as age, sex, appearance, and humor did not have a great impact on the evaluation of the professors • The type of evaluation of the students There is a significant difference based on gender and educational level (*p* < 0.05).
4	Sepahi. V et al. 2016 [17]	Iran	descriptive analytical study	Examining the factors affecting the evaluation of professors from the students’ point of view and its relationship with the academic status	554 students, simple random sampling, data collection tool, researcher-made questionnaire with 37 questions including 5 areas: teacher’s teaching skills, teacher’s personal characteristics, student’s personal characteristics and attitude, physical characteristics and lesson delivery time, and characteristics of the evaluation process in University	• There was no significant relationship found between the factors affecting teaching skills and the personal characteristics of students on their evaluation of professors by their academic status. • The results showed that personal characteristics, the attitude of students, and the timing of class delivery, from the students’ perspective, are factors that have a significant relationship with the academic status of students in their evaluation of professors (*p* = 0.037 and *p* = 0.040).
5	Soriano. G et al. 2017 [18]	Philippines	descriptive-survey	Identifying nursing students’ and clinical instructors’ perceptions of the characteristics of a good clinical professor and whether there are differences and commonalities between these two groups.	80 fourth-year nursing students from College of Nursing, purposive sampling, using the Nursing Clinical Professors Effectiveness Questionnaire (NCTEI) prepared by Knox and Morgan (1987)	• The professor’s teaching ability got the least points and personality traits got the most points from the students’ point of view. • The clinical teaching behaviors with the highest scores by the students were placed in the personality category. • The top three educational behaviors of a professor include; It was discipline, self-confidence, dynamism and being energetic. Other top items include; Interpersonal relationships and nursing competence.
6	Spark. MJ et al. 2017 [19]	Australia	Cross-sectional study	Examining the characteristics of La Trobe pharmacy students (Australia) as characteristics of a good lecturer (faculty member) and comparing the findings with undergraduate pharmacy students at Cardiff University, Wales, England (UK)	183 students, a 22-question questionnaire prepared by the Cardiff faculty for Latrobe University students included 22 questions describing the characteristics of a good lecturer using a 5-point Likert scale.	• Pharmacy students believed that good instructors (faculty) provided clear guidelines and evaluation criteria, were enthusiastic about teaching, encouraged students to do their best, motivated students to learn, were available for support, and made teaching sessions They started on time. They also provided timely feedback and demonstrated the relevance of materials to pharmaceuticals. • Australian and UK pharmacy graduates in this study shared similar views on most aspects of positive faculty characteristics.
7	Kavosi. Z et al. 2017 [20]	Iran	descriptive-analytical cross-sectional study	Evaluation of existing evaluation criteria in the form of evaluation of students from the professors of Shiraz University of Medical Sciences	240 students of Shiraz University of Medical Sciences, stratified sampling according to the size of the population, two-part questionnaire, the first part including the demographic characteristics of the participants and the second part including questions related to the six main items in the evaluation form of professors; “Attracting students’ attention during teaching”, “Using interactive and new teaching techniques”, “Ability to make the subject understandable and motivating, “Timeliness”, “Proper communication” and “Proper planning”	• Of the six evaluation criteria, “attracting students’ attention” had the highest weight, followed by “using interactive and innovative teaching methods,” “ability to understand the subject and create motivation,” " punctuality,” “appropriate communication,” and “appropriate planning.”
8	Hamedi-Asl. P et al. 2018 [21]	Iran	descriptive - cross-sectional	Determining the effective factors on the professor’s evaluation score from the students’ point of view at Jahrom University of Medical Sciences in 2016	287 students of various fields working in Jahrom University of Medical Sciences, required information using the demographic profile form and the standard questionnaire of factors affecting students’ opinions about professors’ evaluation	• There is a significant difference between male and female students regarding the importance of teaching skills in teacher evaluation (*p* = 0.001). Female students scored higher than males in this field. • A significant difference was observed between the students of different semesters regarding the importance of individual characteristics, teaching skills, communication skills and educational rules in the evaluation of professors (p 0.05). • The most important areas affecting students’ evaluation of better professors included teaching skills, communication skills, educational rules, and personal characteristics. • 75.46% of students believed that teaching skills are the most important area influencing the evaluation of professors. Communication skills (67.99%), educational rules (63.92%) and personal characteristics (49.58%) were ranked second to fourth. • The findings of the research showed that teaching skills are the most important factors for students in evaluating professors, and communication skills, educational rules, and individual characteristics are other important factors in this field.
9	Shareinia. H et al. 2018 [22]	Iran	cross-sectional study	Determining the relationship between social and academic cohesion of students with the evaluation of professors of Gonabad University of Medical Sciences in 2016	307 continuous undergraduate students of Gonabad University of Medical Sciences in 2016, selected by stratified random method, tools; Demographic information questionnaire, standard tool of academic and social cohesion, as well as academic faculty performance quality questionnaire	• Among the dimensions of social cohesion, the highest score (29.4 ± 08.59) was related to peer group interactions, and among the dimensions of academic cohesion, the highest score (26.4 ± 34.32) was related to academic and intellectual progress. • Among the evaluation dimensions of professors’ performance quality, the highest score was related to class management (27.4 ± 63.85). • Pearson’s correlation test showed that there is a direct and significant relationship between the overall score of social and academic cohesion and the overall score of evaluating the quality of professors’ performance (*p* = 0.04, *p* < 0.001, *r* = 0.11 and *r* = 0.53, respectively). • According to the results of the linear regression test, for each increase in social cohesion, the evaluation score of professors increased by 0.11 and for academic cohesion, the evaluation score of professors increased by 0.53.
10	Yaghoubi. M et al. 2018 [23]	Iran	cross-sectional study	Investigating the factors affecting the educational evaluation of professors from the point of view of professors and students	The sample studied in the factor analysis phase was 84 students and in the cross-sectional phase 344 students of the University of Military Medical Sciences in Tehran. the tool used; Educational evaluation questionnaire of professors obtained from confirmatory factor analysis	• Based on factor analysis, all dimensions of professors’ educational evaluation had a significant effect at the confidence level of 99%. • The standard regression coefficient was 0.48 in teaching quality, 0.43 in individual characteristics, 0.29 in educational rules and 0.37 in professional characteristics. • Among the dimensions of educational evaluation of professors from the students’ point of view, the dimension of personal characteristics (3.66 ± 0.82) had the highest mean. • The mean and standard deviation of the total educational evaluation of professors was found to be (3.37 ± 0.61). • Friedman’s test showed that the dimension of individual characteristics has the highest rank among other dimensions and the average difference between the dimensions is statistically significant.
11	Ganbari. S et al. 2018 [24]	Iran	descriptive and correlation	Investigating the effect of evaluation of professors by students on the teaching quality of faculty members	stratified random sampling method according to the size of each class based on the educational level of 195 students, data collection tools, two standard evaluation questionnaires of professors’ performance and teaching quality	• The dimensions of evaluation of professors: teaching method, mastery and academic ability, and personal and social characteristics of the professor have a positive and significant effect on the teaching quality of faculty members.
12	Heidari. AA et al. 2018 [25]	Iran	qualitative Study	Explaining the opinions of the assistants regarding the teaching of the professors of Mashhad Medical School	639 assistants, collecting data with survey forms based on Likert scale and an open question and finally analyzing the views, perceptions and experiences of assistants in two categories with positive and negative opinions.	• The themes that emerged in this study regarding the teaching of professors included professional qualifications (with subcategories of academic competence, interest, and practical skills) and personal characteristics (with subcategories of personal qualities and ethical behavior).
13	El-Sayed. M et al. 2018 [26]	Oman	cross-sectional	Investigating medical students’ understanding of teaching evaluation feedback and investigating medical students’ beliefs about the importance and usefulness of feedback at the end of the course	192 pre-clinical students in Oman Medical College, a 26-question questionnaire to evaluate medical students’ perception of professors’ teaching evaluation, the four main topics evaluated in the questionnaire include; The usefulness of teaching evaluation by faculty members, the usefulness of teaching evaluation by college management, knowledge of the teaching evaluation process and valid criteria for evaluating professors.	• The following criteria are necessary for effective evaluation of professors: expertise in content (71.35%), ability to attract students’ attention (83.85%), promotion of critical thinking (77.08%), effective use of audiovisual equipment (78.65%), encouragement and motivation of students (77.08%), and demonstration of participant enjoyment (81.77%). • Most students felt that professors use student feedback information to improve the course (58.85%), to amend evaluation methods and procedures (54.16%), and to promote learner-centered teaching (41.65%). • They strongly felt (60.40%) that teaching evaluation should be done mid-semester rather than at the end of the academic year.
14	Arasteh. MT et al. 2018 [27]	Iran	cross-sectional	Determining the conformity of professors’ self-evaluation results and the evaluation results of other groups	Using 43 questions in the form of 5 questionnaires, 120 faculty members were evaluated by students, colleagues, faculty members and the faculty dean. The research community is all faculty members (as lecturers) and students of different faculties of this university, 1100 students (from 4 levels of doctorate, master’s degree, bachelor’s and associate degree) and 120 faculty members.	• The important points of attention of the students in the evaluation of the professors included these items; The use of educational aids within the scope of the facilities and appropriate to the type of course, the ability to manage the classroom, the suitability of the taught content with the student’s educational needs, encouraging students to learn, appropriate social behavior and mutual respect with students, allocating enough time to answer questions. students, forcing students to participate in discussions, the ability to express and understand course objectives, introducing suitable resources, fully explaining the objectives of the course, presenting lessons in a practical manner with suitable examples, fully mastering the course content, observing cultural and ethical issues in the classroom Determining how to evaluate from the beginning of teaching, motivating students to continue their studies, regular attendance and proper use of class time, evaluating students through appropriate questions during the semester, paying attention to students’ attendance and absence, presenting lesson plans and observing class time.
15	Rahimi Moghadam. S et al. 2019 [28]	Iran	cross-sectional descriptive and analytical study	Examining the evaluation priorities of professors from the perspective of students of Neishabur University of Medical Sciences	140 students of Neishabur Faculty of Medical Sciences, according to census. Using the questionnaire made in the study of Heydari et al.	• According to the students, the factors of a good professor included these items; mastery of the lesson subject in the “teaching skills section”, the way of expressing and conveying concepts and understanding the material in the “individual characteristics section”, respect for the student in the “communication skills section”, the exact start and end time of the class in the “law and regulation compliance area” And a comprehensive and detailed exam at the end of the academic semester in the “Evaluation Skills Section”. • According to the students, there was no significant difference between the 5 investigated areas. • There was no significant relationship between grade point average and any of the evaluation areas. • There was a significant difference between gender and the two areas of compliance with rules and regulations and teaching skills.
16	Myerholtz. L et al. 2019 [29]	USA	descriptive	Existing and ideal characteristics of faculty teaching evaluation systems from the perspective of key stakeholders: faculty, assistants, and residency program directors (PDs).	126 samples were used from two qualitative approaches, confidential semi-structured telephone interviews and anonymous online survey of assistants.	• Assistants desired practical, real, and continuous faculty evaluation feedback to enhance professional development. • Assistants also noted that feedback should be based on a shared understanding of a faculty member’s skills.
17	Basirat. M et al. 2019 [30]	Iran	descriptive cross-sectional	Evaluation of the professor from the point of view of dental school students during and at the end of the academic semester	All students of the dental school of Gilan University of Medical Sciences in the academic year 94–95, who have been studying for at least two years (120 people), study tool; The evaluation questionnaire of the professors of Gilan University of Medical Sciences at two time points during and at the end of the semester	• There is no significant difference between the average evaluation score during the semester (3.41 ± 0.38) and at the end of the semester (3.3 ± 0.24) (*p* = 0.206). • A significant difference was observed in the average score during the semester and at the end of the academic semester of the evaluation of the professors in the items of the professor’s scientific mastery, the way of presenting the material, observing the sequence and priority of the material, the professor’s punctuality, and the ability to control and manage the class (*p* > 0.05). • There is no significant difference between the student’s academic year and the average evaluation score of the professors.
18	Stroud. L et al. 2020 [31]	Canada	descriptive	Investigating the effect of gender bias in the evaluation of the assistants from the professors’ teaching in 3 clinical departments	1560 teaching assistants evaluated faculty in various clinical areas using the Teaching Effectiveness Evaluation (RATE) form at the end of each rotation.	• The effects of gender were different in the sectors. In internal medicine (38.5% female faculty members), no significant gender effect was observed. In surgery (16.2% female) and family medicine (53.0% female), male faculty members received significantly higher scores than female faculty members. In surgery, this was done by male residents who gave higher ratings to male faculty (4.46 vs. 4.26, *p* < 0.001). In family medicine, this was done because male faculty received ratings regardless of gender.
19	Arrona-Palacios. A et al. 2020 [32]	Mexico	descriptive	Investigating the effect of professors’ gender based on student evaluation of teaching	103,833 faculty students (first to last semester) from a private university in Mexico evaluated 5,083 faculty members. Questionnaire (ECOA Encuesta de Opinion de Alumnos) was used.	• Regardless of gender, students evaluate the teaching performance of their professors based on specific criteria, however, in an overall evaluation, students preferred male professors over their female counterparts with a small difference.
20	Griffith. AL A et al. 2021 [33]	USA	descriptive and analytical study	Investigating the effect of professors’ gender on students’ grades	Samples of students related to 2640 professors of a large public university, examination of data on professors’ gender, their contract status, and students’ grades.	• Students whose classes were taught by a female instructor with job uncertainty status scored higher. • These higher scores indicate more lenient grading rather than better preparation for subsequent courses. • Students who attend classes with male instructors, there is no significant difference between the instructor’s rank in the grades received.
21	Patacsil F. F et al. 2022 [1]	Philippines	descriptive	Creating a model to predict the performance of faculty members using associative law based on the evaluation form available by PSU (Pangasinan State University) to evaluate faculty members.	Information of 15,548 students was collected from PSU online portal. Send questionnaires to each student’s portal so that they can evaluate the performance of their instructors.	“Teaching the subject/subject well”, “explains simply” can be used to evaluate the teacher’s performance.

The study was carried out on 130,187 student samples from studies conducted in Iran, the United States, Spain, Canada, Mexico, Australia, Oman, and the Philippines. Based on the findings of the reviewed articles, the factors affecting the evaluation of teachers’ medical universities from the students’ point of view were identified and these factors were categorized by the researchers, which can be introduced in 6 dimensions and 53 components. The 6 dimensions include: individual and professional characteristics of the educational system with 26 components, attitude within the educational system with 7 components, educational programs and guides with 3 components, teaching methodology with 7 components, internal coherence of educational resources with 4 components, and evaluation system information with 6 components. The dimension of individual and professional characteristics of the educational system, which has more components than other dimensions, and more studies pointed out these components. The most important components of this dimension include: individual and social characteristics, teachers’ scientific mastery, teaching quality, communication skills, ability to attract students’ attention, and classroom management. In the dimension of attitude within the educational system, respect for students, teacher’s attitude, support for students were among the most important components of this dimension. The dimension of educational programs and guides consist of linking content with different career, matching competencies with career development and appropriate planning. The teaching methodology dimension includes important components, encouraging and giving motivation, involving students in discussions, presenting lessons in a practical way, using interactive and innovative teaching methods. The most important components of the internal coherence of educational resources include helping to identify related sources and books, organizing the content, fully explaining lesson objectives and presenting lesson plan. In the dimension of evaluation system information, evaluation skills, appropriateness between course content and exam questions, diversity of student learning evaluation procedures, providing timely feedback are important components. The dimensions and components extracted from the studies are detailed in Table 2.

**Table 2:** Classification of evaluation dimensions and its components

Evaluation dimensions	Subset components of each dimension
1. Individual and professional characteristics of the educational system [1, 4, 15, 17–33]	Class management, teachers scientific mastery, how to present the material, respecting the sequence and priority of the material, attendance on time, the ability to control and manage the class, teaching quality, individual and social characteristics, educational rules, professional characteristics, ability to understand the material, communication skills, Age, gender, appearance, humor, teaching skills, ability to attract student’s attention, promotion of critical thinking, ability to understand the subject, personality, educational clinical behavior, interpersonal relationships, effective use of audio-visual equipment, lesson delivery time, teachers rank
2. Attitude within the educational system [16, 19, 25, 27]	Respect for students, interest in the subject by the teacher, compliance with cultural and ethical issues, allocating time to answer students’ questions, paying attention to attendance and absence, teacher’s attitude, supporting students
3. Educational programs and guides [18, 20, 27]	Linking content with different career aspects, matching competencies with career development, appropriate planning
4. Teaching methodology [4, 16, 19, 20, 24, 26, 27]	Encouraging and giving motivation, showing the pleasure of the participant, involving students in discussions, presenting lessons in a practical way with examples, using interactive and innovative teaching methods, observing the appropriateness of the content with the comprehensive scientific level, teaching method
5. Internal coherence of educational resources [16, 27]	Helping to identify related sources and books, organizing and presenting content, fully explaining lesson objectives and presenting lesson plans
6. Evaluation system information [15, 16, 19, 27, 28]	Evaluation skill, appropriateness between course content and exam questions, holding a progress evaluation test, adaptation of the evaluation system used to tasks, diversity of student learning evaluation procedures, providing timely feedback

## Discussion

This systematic review study was conducted with the aim of investigating the factors affecting the evaluation of teachers’ medical universities from the perspective of students.

The results of the reviewed articles showed that these factors can be categorized into six dimensions: Individual and professional characteristics of the educational system, attitude within the educational system, educational programs and guides, teaching methodology, internal consistency of educational resources, and evaluation system information. Each dimension and extracted components were discussed with other studies. Which will be explained as follows.

### Individual and professional characteristics of the educational system

Siamian’s et al., 2013 study showed that “proficiency in expression” is one of the most important characteristics of a good teacher [34]. On the other hand, the El-Sayed et al., 2018 study, many students believed that the personality and attractiveness of faculty members affect their ranking [26]. However, this result was not found in Amr’s et al, 2012 study [35], which may be because more than 90% of the students in the El-Sayed study [26]were female. Mohammadi et al., 2015 believed that interaction between students and other students, faculty members, and staff in the university environment increases their satisfaction and interest and affects the evaluation of students’ performance by teachers [36]. Similar results were found in a study conducted on Omani nursing students, which showed that professional competence of mentors was considered the most important evaluation feature, and the relationship between mentors and students was the second most important feature [37]. The results of the Daragahi et al., 2013 demonstrated classroom management received the highest score among the areas of teacher evaluation, followed by course content management, professional role, and teaching and guidance [38]. In some cases, female teachers received better evaluations than male teachers. This difference and lack of agreement between studies suggests that gender bias in student evaluations depends on university background, field, and student body [39]. On the other hand, the findings showed that there is racial bias in the evaluation of teachers, so that people of color, especially black faculty members, were ranked lower than their white counterparts [40]. Evidence showed that older teachers scored lower, but these results disappeared after controlling for other influential factors in evaluation, such as physical appearance and course difficulty [41]. However, in some studies, even after controlling for other influential factors in student evaluation of teachers, it was shown that older teachers receive lower scores [42, 43].

### Attitude within the educational system

Respect is considered a sign of value, and the fact that students feel respected by their teachers may be associated with higher levels of security and comfort in academic participation. Students also reported experiencing or witnessing demeaning statements, nonverbal disregard, and differential treatment by instructors [44]. The results of the Malekshahi et al., 2011 study showed that students prioritize respect for the student as a very important factor in evaluation [45]. The results of some researches also showed that the availability of the teacher and the time spent on solving students’ educational problems are influential factors in the evaluation of teachers, and students believed that such teachers would receive better grades in evaluation [8, 46]. As our study also showed, the type of behavior, attitude, and approach of the educational system towards students in the evaluation of this group is very important and is one of the pillars that students learn from.

### Educational programs and guides

Students considered proper planning as one of the dimensions of evaluating teachers. The results indicated that students consider seriousness, planning, and organization of topics to be highly important [34, 47]. Furthermore, it has been demonstrated that teachers proficiency in lesson planning and organization plays a significant role in evaluating students [48]. This study also showed that expressing the link between topics and various professional aspects, required professional competencies, and appropriate planning for courses are among the factors that have an impact on evaluating teachers by students in this dimension.

### Teaching methodology

In Lopez’s et al., 2015 study, it is stated that teachers should decide on what methods to use in the classroom (individual, group, collaborative, etc.), what teaching and learning strategies to implement, what types of social relationships and groupings to create with their students, what types of activities to propose and in what order, and how to deal with the diversity of students [16]. The use of innovative and different teaching techniques instead of just lecturing leads to better evaluation of teachers by students, and having a lot of knowledge does not necessarily make someone a good teacher [20]. One of the dimensions of teachers evaluation by students is the use of instructional aids relevant to the subject [47]. In today’s age, a teacher must be aware, prepared, and familiar with the latest science. Preparedness does not mean accumulating information, but rather educational and research abilities, and awareness of innovative teaching methods is one of the necessities of this key role [49]. In our study, in addition to the mentioned cases, it was shown that encouraging and motivating students, encouraging them to participate in discussions and presenting lessons practically, and considering the proportionality of the materials to the students level of knowledge are taken into account in evaluating teachers.

### Internal coherence of teaching resources

Educational resources encompass a wide range of techniques, strategies, tools, and materials, from white/blackboards and markers to videos and the use of the internet [16]. A study showed that approximately 88% of the surveyed students considered the organization of instructional materials, fairness in grading, and learning of the taught materials to be very important in evaluating teachers [50]. On the other hand, helping students identify relevant course materials and textbooks by teachers and providing a comprehensive description of lesson objectives and presenting a lesson plan are also considered important by students.

### Evaluation system information

The term “evaluation system” refers to a systematic set of processes that collects, analyzes, and interprets relevant information used to measure or describe each aspect of the educational reality, and based on this description, develops a value judgment using a criterion or model as a decision-making basis. Value judgments are made about various aspects that affect the teaching-learning process and confirm the skills acquired [51]. Skills in evaluation by teachers include the alignment of course content and exam questions, conducting progress evaluation tests, diversity in learning evaluation methods for students, and timely feedback to students, among other things.

## Conclusion

The ultimate goal of the higher education system is to provide conditions for students to acquire knowledge, skills, and attitudes, and the main responsibility lies with the members of the faculty. Therefore, the need for continuous evaluation by stakeholders leads to the promotion and excellence of the university, and considering the students’ perspective as part of the university’s educational process is recommended to address deficiencies and improve education. The study results showed that from the students’ point of view, the individual and professional characteristics of the educational system, attitude within the educational system, educational programs and guides, teaching methodology, internal coherence of educational resources, and evaluation system information are factors affecting the evaluation of teachers. The results obtained in this study can be used in the selection of teachers and faculty members of universities because these results are derived from the factors affecting the evaluation of teachers from the students’ point of view. Also, by holding practical educational workshops that take into account the dimensions and components extracted in this study, it is possible to increase the quality of teaching and learning.

### Weaknesses, strengths and limitations of the study

The strength of this study is the extensive review of databases and search engines, as well as the high sample size of the studies examined. The weakness of this study is the failure to consider articles in languages other than English and Persian, as well as only considering the students’ perspective and not taking into account the teachers’ perspective regarding the evaluation of teachers, which could be addressed in another project. It is recommended that future studies also consider intervention research. One limitation of the study was the lack of access to the full text of some articles.

### Electronic supplementary material

Below is the link to the electronic supplementary material.

Supplementary Material 1

Supplementary Material 2

## Data Availability

The datasets generated and/or analyzed during the current study are available from the corresponding author on reasonable request.

## References

[1] Patacsil FF, Cenas PV, Roaring BF, Parrone JM. Evaluating Pangasinan State University Faculty Performance using associative rule analysis. Int J Inform Educ Technol. 2022;12(1).

[2] Al-Sudani D, Al-Abbas F, Al-Bannawi Z, Al-Ramadhan A (2013). Professional attitudes and behaviors acquired during undergraduate education in the College of Dentistry, King Saud University. Saudi Dent J.

[3] Paulsen MB (2002). Evaluating teaching performance. New Dir Institutional Res.

[4] Yaminfirooz M, Esmaeili T (2017). What Criteria do students use to EvaluateTheir teachers?. Bimon Educ Strategies Med Sci.

[5] Alibeygi AH, Barani S, Dehkordi MK (2019). Designing a comprehensive model for teaching quality evaluation of faculty members: the case of Razi University. Curriculum Plann.

[6] Yaghoubi M, Salimi M, Karamali M, Ehsani-Chimeh E (2019). Factors affecting the evaluation of teachers by systematic and Delphi methods in the military university in Tehran. J Mil Med.

[7] Nobakht M, RoudbariI M (2013). Students assess the quality of instruction at the University of Tehran. J Teb Va Tazkiyeh.

[8] Vakili A, Hajaghajani S, Rashidy-Pour A, Ghorbani R. An investigation of factors influencing student evaluation of teacher performance: A comprehensive study in Semnan University of Medical Sciences. Koomesh. 2011:93–103.

[9] Boring A, Philippe A (2021). Reducing discrimination in the field: evidence from an awareness raising intervention targeting gender biases in student evaluations of teaching. J Public Econ.

[10] Mengel F, Sauermann J, Zölitz U (2019). Gender bias in teaching evaluations. J Eur Econ Assoc.

[11] Wiley C (2019). Standardised module evaluation surveys in UK higher education: establishing students’ perspectives. Stud Educational Evaluation.

[12] Hills SB, Naegle N, Bartkus KR (2009). How important are items on a student evaluation? A study of item salience. J Educ Bus.

[13] Bagherian Far M, Nasr Esfahani AR, Ahanchian MR (2020). A comparative study of assessment procedures in Universities of Iran and World Top universities. J New Thoughts Educ.

[14] Tricco AC, Lillie E, Zarin W, O’Brien KK, Colquhoun H, Levac D (2018). PRISMA extension for scoping reviews (PRISMA-ScR): checklist and explanation. Ann Intern Med.

[15] Vahabi A, Rahmani S, Rostami S, Vahabi B, Hosseini M, Roshani D (2015). Factors affecting teacher evaluation scores: the students` viewpoints of Kurdistan University of Medical Sciences. Iran J Med Educ.

[16] López-Cámara A-B, González-López I, de León-Huertas C (2015). Exploratory factor analysis to construct a model of university teaching evaluation indicators/Un análisis factorial exploratorio para la construcción de un modelo de indicadores de evaluación docente universitaria. Cultura Y Educación.

[17] Sepahi V, Khoshay A, POURMIRZA KR, Rostami P. Factors affecting student evaluation of teacher performance and its relationship with educational achievement. 2016.

[18] Soriano G, Aquino M (2017). Characteristics of a good clinical teacher as perceived by nursing students and faculty members in a Philippine University College of Nursing. Int J Nurs Sci.

[19] Spark MJ, Tawil R, O’Brien B, Sutherland-Plozza Z, Charles S, John DN. What are the attributes of good pharmacy faculty (lecturers)? An international comparison of the views of pharmacy undergraduate students from universities in Australia and Wales, UK. Pharm Educ. 2017;17.

[20] Kavosi Z, Nasab SB, Yusefi AR. Professors’ valuation criteria from the perspective of students of Shiraz University of Medical Sciences using Shannon’s Entropy technique in 2016. Strides Dev Med Educ. 2017;14(3).

[21] Hamedi P, Saleh S, Hojati H, Kalani N. The factors affecting the Tenured Faculty Member evaluation score from the perspective of students of Jahrom University of Medical Sciences in 2016. J Res Med Dent Sci. 2018;6(2).

[22] Shareinia H, Jahani M, Rahati J, Sokouti A, Mohammadian B, Najafi S (2018). Relationship between Social and Academic Integration of Students with student evaluation of teacher performance in Gonabad University of Medical Sciences, Gonabad, Iran in 2016. J Med Educ Dev.

[23] Yaghoubi M, Salimi M (2018). Determining the factors affecting faculties’ educational evaluation in a military university of medical sciences in Tehran, Iran. J Military Med.

[24] Ganbari S, Soltanzadeh V (2018). Improving the quality of teaching in the light of the evaluation of professors: reflectively on students’ perspective. J Res Teach.

[25] Heidari AA, Khooei A, Dadgarmoghadam M (2018). Content analysis of educational assistants views regarding the evaluation of Mashhad University of Medical Sciences’ professors in educational clinical departments: a qualitative study. Future Med Educ J.

[26] El-Sayed M, Simon MA, El-Wasify M, Nambiar V (2018). Medical students’ perception of teaching evaluation and feedback: a study at Oman Medical College. Middle East Curr Psychiatry.

[27] Arasteh MT, Pouragha B, Norouzinia R (2018). Studying the conformity of self-assessment results of higher education lecturers with the assessment by others. Int J Pharm Res.

[28] Rahimi Moghadam, Hosseini MS, Fekri N, Emkani M (2019). Survey of the priorities of teachers evaluation and effective factors from students viewpoint of Neyshabur University of Medical Sciences. Med Educ.

[29] Myerholtz L, Reid A, Baker H, Rollins L, Page C (2019). Residency faculty teaching evaluation: what do faculty, residents, and program directors want?. Fam Med.

[30] Basirat M, Motevasseli S, Mirfarhadi N, Taheri M (2019). Dentistry Students´ viewpoints about evaluation of Faculty members during and at the end of Semester in 2016. Res Med Educ.

[31] Stroud L, Freeman R, Kulasegaram K, Cil TD, Ginsburg S (2020). Gender effects in assessment of clinical teaching: does concordance matter?. J Graduate Med Educ.

[32] Arrona-Palacios A, Okoye K, Camacho-Zuñiga C, Hammout N, Luttmann-Nakamura E, Hosseini S (2020). Does professors’ gender impact how students evaluate their teaching and the recommendations for the best professor?. Heliyon.

[33] Griffith AL, Sovero V (2021). Under pressure: how faculty gender and contract uncertainty impact students’ grades. Econ Educ Rev.

[34] Siamian H, Bala Ghafari A, Aligolbandi K, Seyyede Fereshteh Reza Nezhad SF, Sharifi Nick M, Shahrabi A (2013). Characteristics of a good university lecturer according to students. J Mazandaran Univ Med Sci.

[35] Amr M, Al Saeed U, Shams T (2012). Medical students’ perceptions of teaching evaluation in psychiatry. Basic Res J Educ Res Rev.

[36] Mohammadi M, Ghatrei H (2015). The role of students’ Social and Academic Integration in their evaluation of faculties’ Educational Performance Quality in Shiraz University of Medical Sciences. Iran J Med Educ.

[37] Madhavanprabhakaran GK, Shukri RK, Hayudini J, Narayanan SK (2013). Undergraduate nursing students’ perception of effective clinical instructor: Oman. Int J Nurs Sci.

[38] Dargahi H, Mohammadzadeh N (2013). Faculty Members’ evaluation by students: valid or invalid. Iran J Med Educ.

[39] Boring A, Ottoboni K. Student evaluations of teaching (mostly) do not measure teaching effectiveness. ScienceOpen Res. 2016.

[40] Chávez K, Mitchell KM (2020). Exploring bias in student evaluations: gender, race, and ethnicity. PS: Political Sci Politics.

[41] Stonebraker RJ, Stone GS (2015). Too old to teach? The effect of age on college and university professors. Res High Educt.

[42] Sohr-Preston SL, Boswell SS, McCaleb K, Robertson D. Professor gender, age, and hotness in influencing college students’ generation and interpretation of professor ratings. 2016.

[43] Wilson JH, Beyer D, Monteiro H (2014). Professor age affects student ratings: Halo effect for younger teachers. Coll Teach.

[44] Liang CT, Rocchino GH, Gutekunst MH, Paulvin C, Melo Li K, Elam-Snowden T (2020). Perspectives of respect, teacher–student relationships, and school climate among boys of color: a multifocus group study. Psychol Men Masculinities.

[45] malekshahi f (2011). Shaikhian a, tarrahi mj. Attitude of students of Lorestan Medical Science University towards priorities in teachers Assessment Iranian. J Nurs Res.

[46] Kerman Saravi F, Navidian A, Navabi Rigi SD. Nursing Student and Teachers’ Viewpoints toward Priorities in Teachers Evaluation. Iran J Nurs (2008–5923). 2011;24(72).

[47] Zare Bidaki M, Rajabpour Sanati A, Hashemian S, Rajai Ghannad F, Nadjafi Semnani M (2014). A survey on students’ attitude toward teachers’ educational characteristics in Birjand University of Medical Sciences in 2014. J Med Educ Dev.

[48] Aliasgharpour M, Monjamed Z, Bahrani N. Factors affecting students’ evaluation of teachers: Comparing viewpoints of teachers and students. Iran J Med Educ. 2010;10(2).

[49] Jirovec RLRC, Alvarez AR (2014). Course evaluations. J Soc Work Educ.

[50] Crumbley L, Henry BK, Kratchman SH. Students’ perceptions of the evaluation of college teaching. Quality assurance in Education; 2001.

[51] Zabalza MÁ, Beraza MÁZ. Competencias docentes del profesorado universitario: calidad y desarrollo profesional. Narcea Ediciones; 2003.

