# Supplementary material for: Investigating factors affecting the evaluation of teachers’ medical universities from the students’ point of view: a systematic review
Source: BMC Med Educ. 2024 Feb 23;24:187. doi: 10.1186/s12909-024-05161-3 (PMC10893686; doi:10.1186/s12909-024-05161-3)
Supplement: Supplementary file 1 — Supplementary Material 1 [file 12909_2024_5161_MOESM1_ESM.docx]

Database search strategy

| **Database** | **Strategy** | **Number of articles** |
| --- | --- | --- |
| **Web of Science** | ("Evaluation" OR "assessment" OR "estimate" OR "appraisement" OR "appraisal") AND ("Faculty member" OR "professor") AND ("University" OR "College") | 532 |
| **Pubmed** | Search: ("Evaluation"[Title/Abstract] OR "assessment"[Title/Abstract] OR "estimate"[Title/Abstract] OR "appraisement"[Title/Abstract] OR "appraisal"[Title/Abstract]) AND ("Faculty member"[Title/Abstract] OR "professor"[Title/Abstract]) AND ("University"[Title/Abstract] OR "College"[Title/Abstract]) Filters: Full text, English, from 2014 - 2022 | 185 |
| **Cochrane** | (Evaluation OR assessment OR estimate OR appraisement OR appraisal) AND (Faculty member OR professor ) AND (University OR College) | 328 |
| **Science direct** | ("Evaluation" OR "assessment" OR "estimate" OR "appraisement" OR "appraisal") AND ("Faculty member" OR "professor") AND ("University" OR "College") | 391 |
| **Scopus** | ("Evaluation" OR "assessment" OR "estimate" OR "appraisement" OR "appraisal") AND ("Faculty member" OR "professor") AND ("University" OR "College") | 2249 |
| **Google scholar** | Evaluation OR assessment OR estimate OR appraisement OR appraisal AND  Faculty member OR professor AND  University OR College | 564000 |
| **Iran doc**  **)Iranian database(** | Evaluation of professor, Evaluation of Faculty member, assessment of professor, assessment of Faculty member | 55 |
| **Sid**  **)Iranian database(** | Evaluation of professor, Evaluation of Faculty member, assessment of professor, assessment of Faculty member | 51 |
| **Magiran**  **)Iranian database(** | Evaluation of professor, Evaluation of Faculty member, assessment of professor, assessment of Faculty member | 158 |
