# Supplementary material for: Investigating factors affecting the evaluation of teachers’ medical universities from the students’ point of view: a systematic review
Source: BMC Med Educ. 2024 Feb 23;24:187. doi: 10.1186/s12909-024-05161-3 (PMC10893686; doi:10.1186/s12909-024-05161-3)
Supplement: Supplementary file 2 — Supplementary Material 2 [file 12909_2024_5161_MOESM2_ESM.docx]

STROBE and COREQ CHECHLIST

| article | | Shareinia. H | **Basirat. M** | **Yaghoubi. M** | **Yaminfirooz. M** | **Sepahi. V** | **Ganbari. S** | **Rahimi Moghadam. S** | **Vahabi. A** | Hamedi-Asl. P | MyerholtZ. L | Patacsil F. F | Stroud. L | El-Sayed. M | **Arasteh. MT** | Griffith. AL | Arrona-Palacios. A | Kavosi. Z | Soriano. G | AnaBelén López-Cámara 2015 | Spark. MJ |
| --- | --- | --- | --- | --- | --- | --- | --- | --- | --- | --- | --- | --- | --- | --- | --- | --- | --- | --- | --- | --- | --- |
| Title and abstract | a | 2 | 2 | 2 | 2 | 2 | 2 | 2 | 2 | 2 | 2 | 2 | 2 | 2 | 2 | 2 | 2 | 2 | 2 | 2 | 2 |
|  | b | 2 | 2 | 2 | 2 | 2 | 2 | 2 | 2 | 2 | 2 | 2 | 2 | 2 | 2 | 2 | 2 | 2 | 2 | 2 | 2 |
| Background  /rationale | | 2 | 2 | 2 | 2 | 2 | 2 | 2 | 2 | 2 | 2 | 2 | 2 | 2 | 2 | 2 | 2 | 2 | 2 | 2 | 2 |
| Objectives | | 2 | 2 | 2 | 2 | 2 | 2 | 2 | 2 | 2 | 2 | 2 | 2 | 2 | 2 | 2 | 2 | 2 | 2 | 2 | 2 |
| Study design | | 2 | 2 | 2 | 2 | 2 | 2 | 2 | 2 | 2 | 2 | 2 | 2 | 2 | 2 | 1 | 2 | 2 | 2 | 2 | 2 |
| Setting | | 2 | 2 | 2 | 2 | 2 | 2 | 2 | 2 | 2 | 2 | 2 | 2 | 2 | 2 | 2 | 2 | 2 | 2 | 2 | 2 |
| Participants | a | 2 | 2 | 2 | 2 | 2 | 2 | 2 | 2 | 2 | 2 | 2 | 2 | 2 | 0 | 2 | 2 | 2 | 2 | 0 | 0 |
|  | b | - | - | - | - | - | - | - | - | - | - | - | - | - | - | - | - | - | - | - | - |
| Variables | | 2 | 2 | 2 | 2 | 2 | 2 | 2 | 2 | 2 | 2 | 2 | 2 | 2 | 2 | 2 | 2 | 2 | 2 | 2 | 2 |
| Data sources/ measurement | | 2 | 2 | 2 | 2 | 2 | 2 | 2 | 2 | 2 | 2 | 2 | 2 | 2 | 2 | 1 | 2 | 2 | 2 | 2 | 2 |
| Bias | | 0 | 0 | 0 | 0 | 0 | 0 | 0 | 0 | 0 | 0 | 0 | 0 | 0 | 0 | 0 | 0 | 0 | 0 | 0 | 0 |
| Study size | | 2 | 0 | 0 | 2 | 0 | 2 | 0 | 0 | 2 | 2 | 0 | 0 | 0 | 0 | 0 | 0 | 2 | 0 | 0 | 0 |
| Quantitative variables | | 2 | 2 | 2 | 2 | 2 | 2 | 2 | 2 | 2 | 2 | 2 | 2 | 2 | 2 | 2 | 2 | 2 | 2 | 2 | 2 |
| Statistical methods | a | 2 | 2 | 2 | 2 | 2 | 2 | 2 | 2 | 2 | - | 2 | 2 | 2 | 2 | 2 | 2 | 2 | 2 | 2 | 2 |
|  | b | 2 | 2 | 2 | 2 | 2 | 2 | 2 | 2 | 2 | - | - | 2 | 2 | 2 | 2 | 2 | - | - | - | - |
|  | c | 0 | 0 | 0 | 0 | 0 | 0 | 0 | 0 | 0 | - | - | 0 | 0 | 0 | 0 | 0 | - | - | - | - |
|  | d | 2 | 2 | 2 | 2 | 2 | 2 | 2 | 2 | 2 | - | - | 2 | 2 | 2 | 0 | 2 | - | - | - | - |
|  | e | 0 | 0 | 0 | 0 | 0 | 0 | 0 | 0 | 0 | - | - | 0 | 0 | 0 | 0 | 0 | - | - | - | - |
| Participants | a | 2 | 2 | 2 | 2 | 2 | 2 | 2 | 2 | 2 | 2 | 0 | 2 | 2 | 2 | 0 | 2 | 2 | 2 | 0 | 2 |
|  | b | 0 | 2 | 0 | 0 | 0 | 0 | 0 | 0 | 0 | 0 | 0 | 0 | 0 | 0 | 0 | 0 | 0 | 0 | 0 | 0 |
|  | c | 0 | 0 | 0 | 0 | 0 | 0 | 0 | 0 | 0 | 0 | 0 | 0 | 0 | 0 | 0 | 0 | 0 | 0 | 0 | 0 |
| Descriptive data | a | 2 | 2 | 2 | 2 | 2 | 0 | 2 | 2 | 2 | 2 | 0 | 2 | 2 | 0 | 0 | 2 | 2 | 0 | 0 | 2 |
|  | b | 0 | 0 | 0 | 0 | 0 | 0 | 0 | 0 | 0 | - | - | 0 | 0 | 0 | 0 | 0 | 0 | 0 | 0 | 0 |
|  | c | - | - | - | - | - | - | - | - | - | - | - | - | - | - | - | - | - | - | - | - |
| Outcome data | | 2 | 2 | 2 | 2 | 2 | 2 | 2 | 2 | 2 | 2 | 2 | 2 | 2 | 2 | 2 | 2 | 2 | 2 | 2 | 2 |
| Main results | a | 2 | 2 | 2 | 2 | 2 | 2 | 2 | 2 | 2 | 2 | 2 | 2 | 2 | 2 | 2 | 2 | 2 | 2 | 2 | 2 |
|  | b | 2 | 2 | 2 | 2 | 2 | 2 | 2 | 2 | 2 | 2 | 2 | 2 | 2 | 2 | 2 | 2 | 2 | 2 | 2 | 2 |
|  | c | 2 | 2 | 2 | 2 | 2 | 2 | 2 | 2 | 2 | 2 | 2 | 2 | 2 | 2 | 2 | 2 | 2 | 2 | 2 | 2 |
| Other analyses | | 2 | 2 | 2 | 2 | 2 | 2 | 2 | 2 | 2 | 2 | 2 | 2 | 2 | 2 | 2 | 2 | 2 | 2 | 2 | 2 |
| Key results | | 2 | 2 | 2 | 2 | 2 | 2 | 2 | 2 | 2 | 2 | 2 | 2 | 2 | 2 | 2 | 2 | 2 | 2 | 2 | 2 |
| Limitations | | 0 | 2 | 0 | 0 | 2 | 0 | 2 | 0 | 0 | 0 | 0 | 2 | 0 | 2 | 2 | 0 | 2 | 0 | 0 | 2 |
| Interpretation | | 2 | 2 | 2 | 2 | 2 | 2 | 2 | 2 | 2 | 2 | 2 | 2 | 2 | 2 | 2 | 2 | 2 | 2 | 2 | 2 |
| Generalisability | | 0 | 0 | 2 | 0 | 0 | 0 | 2 | 0 | 0 | 0 | 0 | 0 | 0 | 0 | 0 | 0 | 0 | 0 | 0 | 0 |
| Funding | | 0 | 0 | 0 | 2 | 0 | 0 | 0 | 0 | 0 | 0 | 0 | 0 | 0 | 0 | 0 | 2 | 0 | 0 | 0 | 0 |

The evaluation of articles in this research was done using the STROBE and COREQ checklist.

STROBE checklist.

COREQ checklist was used to evaluate a qualitative study included in the research.

| Heidari. AA | | |
| --- | --- | --- |
| 1 | Interviewer/facilitator | 0 |
| 2 | Credentials | 0 |
| 3 | Occupation | 0 |
| 4 | Gender | 0 |
| 5 | Experience and training | 0 |
| 6 | Relationship established | 0 |
| 7 | Participant knowledge of the interviewer | 1 |
| 8 | Interviewer characteristics | 1 |
| 9 | Methodological orientation and Theory | 1 |
| 10 | Sampling | 1 |
| 11 | Method of approach | 1 |
| 12 | Sample size | 1 |
| 13 | Non-participation | 1 |
| 14 | Setting of data collection | 0 |
| 15 | Presence of nonparticipants | 0 |
| 16 | Description of sample | 1 |
| 17 | Interview guide | 0 |
| 18 | Repeat interviews | 0 |
| 19 | Audio/visual recording | 0 |
| 20 | Field notes | 0 |
| 21 | Duration | 0 |
| 22 | Data saturation | 0 |
| 23 | Transcripts returned | 0 |
| 24 | Number of data coders | 0 |
| 25 | Description of the coding tree | 1 |
| 26 | Derivation of themes | 1 |
| 27 | Software | 0 |
| 28 | Participant checking | 0 |
| 29 | Quotations presented | 0 |
| 30 | Data and findings consistent | 1 |
| 31 | Clarity of major themes | 1 |
| 32 | Clarity of minor themes | 1 |
